# Lesões Plexiformes na Hipertensão Arterial Pulmonar: Estamos Ficando mais Próximos do Manejo com mais Paciência e Rigor?

**DOI:** 10.36660/abc.20200832

**Published:** 2020-09-18

**Authors:** Hugo Hyung Bok Yoo

**Affiliations:** 1 Universidade Estadual Paulista Júlio de Mesquita Filho Faculdade de Medicina Botucatu SP Brasil Universidade Estadual Paulista Júlio de Mesquita Filho Faculdade de Medicina Campus de Botucatu, Botucatu, SP – Brasil

**Keywords:** Roedores, Hipertensão Arterial Pulmonar/fisiopatologia, Resistência Vascular, Vasoconstrição, Insuficiência Cardíaca, Hipóxia

A hipertensão arterial pulmonar (HAP) é uma condição progressiva e potencialmente fatal, caracterizada por pressão arterial pulmonar elevada, remodelamento de pequenos vasos sanguíneos pulmonares e aumento da resistência vascular, levando à insuficiência cardíaca direita. De acordo com o 6º Simpósio Mundial de Hipertensão Pulmonar, a HAP é definida pela elevação concomitante de três parâmetros: pressão arterial pulmonar média (PAPm) >20 mmHg; pressão arterial pulmonar em cunha (PAPC) ≤15 mm Hg; resistência vascular pulmonar (RVP) ≥ 3 unidades de Wood.^1^

A insuficiência cardíaca direita é decorrente de um remodelamento persistente que obstrui e oblitera gradativamente as artérias pulmonares periféricas, causando vasoconstrição e aumentando a pós-carga do ventrículo direito. Muitos esforços têm sido feitos para tratar a HAP. Entretanto, ainda existem poucos tratamentos farmacológicos bem-sucedidos e promissores para essa doença devastadora e, como consequência, a sobrevida relacionada à HAP permanece decepcionante.^2^ Assim, diversas pesquisas sobre a fisiopatologia da hipertensão pulmonar têm se concentrado em uma melhor compreensão do processo de angioproliferação e das lesões plexiformes. O papel do aumento do fluxo sanguíneo no leito vascular pulmonar é considerado um dos principais fatores desencadeantes para o desenvolvimento do remodelamento vascular pulmonar.^2-4^

As lesões plexiformes são estruturas vasculares que ocorrem na HAP idiopática, mas também em outras formas associadas a shunt cardíaco esquerda-direita, doença do tecido conjuntivo, infecção pelo HIV, síndrome CREST, cirrose hepática e esquistossomose.^1-3^ A presença de lesões plexiformes contribui para o desenvolvimento de uma forma grave da HAP, caracterizada por proliferação celular desorganizada nas estruturas glomerulóides, representada por um processo desordenado de angiogênese. Se elas representam sequelas morfológicas de uma pressão intravascular alta anormal, ou contribuem ativamente para o desenvolvimento da doença, continua a ser estudado.^3^

Nas últimas décadas, alguns modelos de roedores foram fundamentais em estudos de hipertensão pulmonar em humanos: modelo de hipóxia crônica; hipertensão pulmonar induzida por monocrotalina (MCT); pneumectomia unilateral esquerda combinada com modelos de MCT ou SU5416 (Sugen).^5-7^ De forma decisiva, eles contribuíram para um melhor entendimento da formação da lesão neointimal da artéria pulmonar periférica.

Entretanto, no modelo de hipóxia crônica, os vasos em remodelamento não apresentam a redução luminal esperada devido ao crescimento intimal e lesões vasculares complexas como observado na HAP humana mais grave.^5^ O modelo de lesão pulmonar por MCT causa alguma disfunção endotelial, mas as lesões vasculares obliterantes observadas na HAP humana grave não são observadas em ratos. Além disso, com a dosagem de MCT, os ratos tendem a morrer frequentemente de toxicidade pulmonar, miocardite e doença hepática veno-oclusiva, em vez de HAP. Em outras palavras, os modelos clássicos não conseguiram induzir a proliferação anormal de células endoteliais capazes de resultar em lesões plexiformes.^6,7^ O modelo de pneumectomia unilateral esquerda aplicando MCT ou Sugen foi realizado para induzir disfunção endotelial. Após cerca de 6 a 8 semanas, os pulmões dos ratos mostraram vasculopatia plexiforme neointimal e artérias pulmonares periféricas obstruídas. Entretanto, a principal limitação desse método é que ele exige habilidades cirúrgicas gerais, ou seja, exige um ambiente de estudo ainda mais restrito para os pesquisadores.^8^

Nesta edição, Gewehr et al.,^9^ utilizando um modelo isolado de MCT, mostraram pela primeira vez a presença de lesões complexas, principalmente do tipo plexiforme, semelhantes às observadas em pacientes com HAP grave. Nesse estudo, o desenvolvimento de muscularização, hipertrofia da camada média e proliferação intimal/neointimal foram caracterizados como alterações iniciais e reversíveis do ponto de vista anatomopatológico. Após 30 dias de efeito da MCT, os ratos já apresentavam hipertrofia ventricular direita. Porém, um processo de remodelamento progressivo, principalmente com lesões plexiformes, mais evidente a partir do 37º dia, possa ser considerado como alterações geralmente irreversíveis. Isso resulta em repercussões hemodinâmicas mais graves e mortalidade precoce, como observado na HAP humana.

Uma peculiaridade que pode justificar os achados das lesões plexiformes no estudo pode estar relacionada ao tempo de observação prolongado de 37 dias, maior do que em estudos anteriores, e a uma análise anatomopatológica pulmonar mais rigorosa. De fato, o aparecimento de lesões vasculares complexas na HAP é dependente do tempo, pois quanto maior o tempo de exposição à MCT, maiores as chances de progressão para lesões plexiformes com sinais de insuficiência cardíaca, como derrame pleural, ascite e congestão hepática. Uma mortalidade de 50% dos ratos foi observada no 37º dia. É difícil determinar se os ratos tratados com MCT morreram devido a HAP ou com HAP.

O que se pode ter certeza é que a HAP é uma doença grave e progressiva e a lesão plexiforme representa um estágio mais avançado e irreversível da HAP. O modelo de MCT sozinho destaca que mais paciência e rigor podem fornecer um maior discernimento no desenvolvimento de estudos de lesões plexiformes. Além disso, ele prevê o teste de novos medicamentos para prevenir lesões ou até mesmo obter a regressão de lesões plexiformes já estabelecidas na HAP.
