# Supplementary material for: Lesões Plexiformes na Hipertensão Arterial Pulmonar: Estamos Ficando mais Próximos do Manejo com mais Paciência e Rigor?
Source: Arq Bras Cardiol. 2020 Sep 18;115(3):491–2. [Article in Portuguese] doi: 10.36660/abc.20200832 (PMC9363101; doi:10.36660/abc.20200832)
Supplement: Translation

# Translation: Plexiform Lesions in Pulmonary Arterial Hypertension: Are we Getting Closer to Manage with More Patience and Rigor?

**Authors:** Hugo Hyung Bok Yoo

**Affiliations:** 1 Brazil Universidade Estadual Paulista Júlio de Mesquita Filho Faculdade de Medicina Campus de Botucatu, Botucatu, SP – Brazil

**Keywords:** Rodentia, Pulmonary Arterial Hypertension/pysiopathology, Vascular Resistance, Heart Failure, Hypoxia

Pulmonary arterial hypertension (PAH) is a progressive and a life-threatening condition characterized by high pulmonary blood pressure, remodeling of small pulmonary blood vessels and increased vascular resistance leading to right heart failure. According to the 6th World Symposium on Pulmonary Hypertension, PAH is defined by concomitant elevation of three parameters: mean pulmonary arterial pressure (mPAP) > 20 mmHg; pulmonary arterial wedge pressure (PAWP) ≤15 mm Hg; pulmonary vascular resistance (PVR) ≥ 3 Wood Units.^1^

The right heart failure is due to a persistent remodeling that gradually obstructs and obliterates the peripheral pulmonary arteries that causes vasoconstriction and increases right ventricle afterload. Many efforts have been made to treat PAH. However, there are still few successful and promising pharmacological treatments for this devasting disease, as a consequence, PAH-related survival remains disappointing.^2^ Thus, several researches on pulmonary hypertension pathophysiology have focused on a better comprehension of the angioproliferation process and plexiform lesions. The role of increased blood flow in the pulmonary vascular bed is considered one of the main triggering factors to the development of pulmonary vascular remodeling.^2-4^

Plexiform lesions are vascular structures that occur in idiopathic PAH, but also in other forms associated with heart deviation from left to right, connective tissue disease, HIV infection, CREST syndrome, liver cirrhosis and Schistosomiasis.^1-3^ The plexiform lesions presence contributes to the development of a severe form of PAH. It is characterized by disorganized cellular proliferation in glomeruloid structures, represented by a disordered angiogenesis process. Whether they represent morphologic sequelae of an abnormal high intravascular pressure or contribute actively to the disease development is still being studied.^3^

In the last decades, some rodent models have been pivotal in human pulmonary hypertension studies: chronic hypoxia model; monocrotaline (MCT) induced pulmonary hypertension; unilateral left pneumonectomy combined with MCT or SU5416 (Sugen) models.^5-7^ Decisively, they have contributed to a better understanding of the peripheral pulmonary artery neointimal lesion formation. Nevertheless, in chronic hypoxia model, the remodeling vessels do not show expected luminal reduction by intimal growth and complex vascular injuries as observed in more severe human PAH.^5^ The MCT lung injury model causes some endothelial dysfunction, but the obliterative vascular lesions observed in human severe PAH are not observed in rats. In addition, with MCT dosing, rats tend to die frequently from pulmonary toxicity, myocarditis and veno-occlusive liver disease rather than due to PAH. In other words, the classic models have failed to induce abnormal endothelial cells proliferation able to result in plexiform lesions.^6,7^ The unilateral left pneumectomy model applying MCT or Sugen was perform to induce endothelial dysfunction. After about 6-8 weeks, rats’ lungs showed plexiform, neointimal vasculopathy and obstructed peripheral pulmonary arteries. However, the main limitation of this method is that it requires general surgical skills, that is, it demands an even more restricted study environment for researchers.^8^

In this issue, Gewehr et al.^9^ in an isolated MCT model showed for the first time the presence of complex lesions, especially plexiform-like ones, similar to those observed in patients with severe PAH. In that study, the development of muscularization, middle layer hypertrophy and intimal/neointimal proliferation were characterized as initial and reversible changes at the anatomopathological point of view. With 30 days under the MCT effect, the rats already presented right ventricular hypertrophy. Although, a progressive remodeling process especially with plexiform lesions, more evident on the 37th day, can be considered as usually irreversible changes. That results in more severe hemodynamic repercussions and early mortality as observed in human PAH. A peculiarity that may justify the plexiform lesions findings in the study may be related to the extended observation time of 37 days, longer than previous studies, and to a more rigorous pulmonary anatomopathological analysis. In fact, the complex vascular lesions appearance in PAH is time-dependent, the longer the time of exposure to MCT, the greater the chances of progression to plexiform lesions with signs of heart failure such as pleural effusion, ascites and liver congestion. Mortality of 50% of rats was observed on the 37th day. Whether MCT-treated rats died due to PAH or with PAH is difficult to determine. What it can be sure is that PAH is a serious progressive disease and the plexiform lesion represents a more advanced and irreversible stage of PAH. The MCT-alone model highlights that more patience and rigor can provide insights in the development of plexiform lesions investigations. In addition, it prospects for testing new drugs to prevent injuries or even regress established plexiform lesions in PAH.
